# The Hydroxypyridinone Iron Chelator DIBI Reduces Bacterial Load and Inflammation in Experimental Lung Infection

**DOI:** 10.3390/biomedicines12071452

**Published:** 2024-06-29

**Authors:** Xiyang Zhang, Rhea Nickerson, Lauren Burton, Ashley Stueck, Bruce Holbein, Zhenyu Cheng, Juan Zhou, Christian Lehmann

**Affiliations:** 1 Department of Anesthesia, Pain Management and Perioperative Medicine, Dalhousie University, Halifax, NS B3H 1X5, Canada; zhangxiyang17@gmail.com (X.Z.); juan.zhou@dal.ca (J.Z.); 2 Department of Anesthesiology, Nanfang Hospital, Southern Medical University, Guangzhou 510515, China; 3 Guangdong Provincial Key Laboratory of Precision Anaesthesia and Perioperative Organ Protection, Guangzhou 510515, China; 4 Department of Microbiology & Immunology, Dalhousie University, Halifax, NS B3H 1X5, Canada; rh395493@dal.ca (R.N.); lr899209@dal.ca (L.B.); beholbein@sympatico.ca (B.H.); zhenyu.cheng@dal.ca (Z.C.); 5 Department of Pathology, Dalhousie University, Halifax, NS B3H 1X5, Canada; ashley.stueck@dal.ca; 6 Department of Physiology & Biophysics, Dalhousie University, Halifax, NS B3H 1X5, Canada; 7 Department of Pharmacology, Dalhousie University, Halifax, NS B3H 4R2, Canada

**Keywords:** lung infection, *Pseudomonas aeruginosa*, iron chelation, inflammation

## Abstract

Iron plays a critical role in lung infections due to its function in the inflammatory immune response but also as an important factor for bacterial growth. Iron chelation represents a potential therapeutic approach to inhibit bacterial growth and pathologically increased pro-inflammatory mediator production. The present study was designed to investigate the impact of the iron chelator DIBI in murine lung infection induced by intratracheal *Pseudomonas aeruginosa* (strain PA14) administration. DIBI is a polymer with a polyvinylpyrrolidone backbone containing nine 3-hydroxy-1-(methacrylamidoethyl)-2-methyl-4(1H) pyridinone (MAHMP) residues per molecule and was given by intraperitoneal injection either as a single dose (80 mg/kg) immediately after PA14 administration or a double dose (second dose 4 h after PA14 administration). The results showed that lung NF-κBp65 levels, as well as levels of various inflammatory cytokines (TNFα, IL-1β, IL-6) both in lung tissue and bronchoalveolar lavage fluid (BALF), were significantly increased 24 h after PA14 administration. Single-dose DIBI did not affect the bacterial load or inflammatory response in the lungs or BALF. However, two doses of DIBI significantly decreased bacterial load, attenuated NF-κBp65 upregulation, reduced inflammatory cytokines production, and relieved lung tissue damage. Our findings support the conclusion that the iron chelator, DIBI, can reduce lung injury induced by *P. aeruginosa*, via its anti-bacterial and anti-inflammatory effects.

## 1. Introduction

As a Gram-negative opportunistic pathogen, *Pseudomonas aeruginosa* is one of the most prevalent pathogens that cause acute and chronic infections in various parts of our body, such as lung, wounds, and urinary tract [1,2]. In recent years, the rate of infections induced by *P. aeruginosa* among hospitalized patients increased significantly. For example, during the past three decades, the rates of hospital-acquired infections caused by *P*. *aeruginosa* was nearly 15%, and the rates for patients with cystic fibrosis (CF) or chronic obstructive pulmonary disease (COPD) was up to 50% [3]. In addition, *P*. *aeruginosa* is the primary pathogen isolated from individuals with hospital-acquired pneumonia, and it is associated with high morbidity and mortality [4]. Moreover, with the increase in multi-drug-resistant strains, eradicating *P. aeruginosa* infections becomes more challenging.

It has been reported that iron dysregulation is an important factor in the maintenance of lung infection induced by *P. aeruginosa* for several reasons [5]. The main reason is that iron is essential for multiple bacterial metabolic pathways and required for host colonization. *P. aeruginosa* utilizes iron acquired from the host to promote growth and increase virulence so as to aggravate the development of lung infection [6]. Secondly, iron is necessary to produce reactive oxygen species (ROS) as part of the immune response to an infection. Excess iron can cause ROS overproduction, which can lead to the damage of healthy cells and aggravation of inflammation [7]. Thirdly, the cells of the immune system require iron to sustain its function, metabolism, and proliferation [8]. However, iron overload can attenuate the phagocytosis of macrophages and affect the function of T lymphocytes, leading to disruption of the immune system [7]. It has been reported that enhancing macrophage iron accumulation promoted acute lung inflammation and oxidative stress, and macrophage ferroportin could serve as a therapeutic target in bacteria-induced acute lung injury [9,10]. Therefore, targeting iron metabolism, specifically iron overload, by using iron chelators is a potential supportive antimicrobial strategy to relieve lung infection caused by *P. aeruginosa*.

Increasing evidence suggests that the hydroxypyridinone iron chelator, DIBI, through depriving microorganisms of bioavailable iron, has the potential to serve as a new anti-infective agent. Allan et al. reported that DIBI showed efficacy in reducing *Staphylococcus aureus* (*S. aureus*) burden in mouse nares comparable to mupirocin [11]. Because of the high sensitivity of *S. aureus*, DIBI was considered as an adjuvant to mupirocin to combat the natural colonization of *S. aureus* isolates. Using minimum inhibitory concentration assay, Ang et al. also demonstrated that DIBI had an inhibitory effect against representative reference strains for Gram-positive and Gram-negative bacteria, such as *S. aureus*, *Acinetobacter baumannii*, and the fungal pathogen *Candida albicans* [12]. In previous experiments, we have demonstrated that DIBI has significant anti-inflammatory effects in lipopolysaccharides (LPS)-induced acute lung injury [13]. In a previously published study by our group, the cytotoxicity of DIBI was evaluated in BALB/C mice for both acute and chronic dosages. The results showed that neither acute nor chronic DIBI administration had cytotoxic effects [14].

However, it is unclear whether DIBI has the same effect against lung infections caused by *P. aeruginosa*. In order to reduce the risk of antimicrobial resistance and explore novel antimicrobial agents or potential adjuvant drugs that can be used in conjunction with existing antibiotics, we investigated the anti-inflammatory and anti-bacterial effects of DIBI in *P. aeruginosa*-induced experimental lung infection in mice.

## 2. Materials and Methods

### 2.1. Bacterial Preparation

*P. aeruginosa* strain PA14 was kept in a freezing medium (50% Luria broth (LB), 50% glycerol) and stored at −80 °C until use. Three days prior to infection experiments, PA14 was streaked on LB agar plates to isolate single colonies and incubated at 37 °C for 16–24 h. Following this, a single colony was selected from the plate and inoculated into 5 mL of LB broth, which was then cultured overnight in a rotating incubator (200 rpm, 37 °C) for at least 18 h. On the third day, the bacterial optical density at 600 nm (OD600) was verified to be in the 5–6 range before the overnight culture was diluted 1:50 in 5 mL of fresh LB broth. The subculture was shaken for approximately 3 h until OD600 reached 1.5–2, indicating that bacteria were in the exponential growth phase. Next, 1 mL of subculture was prepared by centrifugation (5000× *g*, 5 min) and washing in phosphate-buffered saline (PBS). The final concentration was determined by the equation 1 OD600 = 1 × 10^9^ CFU/mL. Bacteria were then diluted in PBS to deliver a dose of 5 × 10^5^ CFU in 40 μL. Meanwhile, bacteria from the final concentration were serially diluted and plated to confirm the accuracy of the dose.

### 2.2. Animals

Age- and weight-matched C57BL/6 male and female mice were purchased from the Jackson Laboratory (Bar Harbor, ME, USA), and they were housed in ventilated plastic cage racks in a pathogen-free room of the Carleton Animal Care Facility, Dalhousie University, Halifax, NS, Canada. Animals were kept on a 12 h light/dark cycle at 21 °C and were acclimatized for one week prior to experiments. Animals were enrolled in experiments at 8–12 weeks of age. Experimental protocols were approved by the University Committee on Laboratory Animals at Dalhousie University under protocol number #21-090 and were performed following the guidelines and standards of the Canadian Council on Animal Care.

### 2.3. Experimental Model

Mice were randomly allocated to four groups as follows: Control + PBS, PA14 + PBS, PA14 + DIBI X1, PA14 + DIBI X2 (*n* = 12/group). Animals were weighed prior to anesthesia, and the induction of anesthesia was accomplished by inhalation of 4–5% isoflurane with oxygen (1 L/min). After 2–3 min induction, once the animals reached the surgical plane of anesthesia (unresponsive to foot pinch), the animals were removed from the nose cone and placed onto a 45° angled platform hanging by the front incisors. The tongue was immobilized, and an otoscope was inserted into the mouth to visualize tracheal opening. Using otoscope guidance, 40 µL of bacterial culture, containing 5 × 10^5^ CFU bacteria, was delivered by pipette just in front of the vocal folds of the tracheal opening. In order to maximize the inhalation of inoculum, after withdrawing the tip, the otoscope was left in the mouth for a few breaths to keep the airway open. After PA14 instillation, the animal was placed back into the cage on a 37 °C heating pad and monitored for 24 h. In the control group (Control + PBS), the mice were given PBS to the tracheal at the same volume of bacterial culture. DIBI (80 mg/kg) was given by intraperitoneal (i.p.) injection either as a single dose immediately after PA14 administration in the PA14 + DIBI X1 group or a double dose (second dose 4 h after PA14 administration) in the PA14 + DIBI X2 group. The mice in the PA14 + PBS group were treated with i.p. PBS once right after PA14 administration.

### 2.4. Clinical Scores and Body Weight Measurement

Mouse physical appearance, posture, righting reflex, respiration rate, activity/behavior and body temperature were scored (each on a scale of 0–3) to assess morbidity after PA14 infection. The clinical scores were recorded 4 h and 24 h after PA14 administration. The weight changes were also recorded at each observation. The clinical scores > 10 and/or weight loss > 15% were considered as criteria for immediate euthanasia.

### 2.5. Bronchoalveolar Lavage Fluid Collection

Twenty-four hours after PA14 infection, mice were anesthetized again. After reaching the surgical plane of anesthesia, blood was collected via cardiac puncture. Then, a total of 1.4 mL of ice-cold PBS with protease inhibitors (Complete tablets, Roche Diagnostic, Basel, Switzerland) was used to perform the bronchoalveolar lavage fluid (BALF) collection. Briefly, after exposure of the trachea, a nick between two of the cartilage rings was created, and a 21-gauge catheter connected to a 3 mL syringe containing protease inhibitors was inserted and immobilized with a nylon string. Lungs were then flushed with 0.7 mL of ice-cold PBS with protease inhibitors. This process was repeated once more. Following BALF collection, lungs were harvested aseptically.

### 2.6. Lung Homogenates

Aseptically-removed lungs were collected in 1 mL sterile PBS with protease inhibitors and put on ice. In a biosafety cabinet, samples were homogenized using a TH115 homogenizer (Omni International, Kennesaw, GA, USA) with sterile probes for 45 s on max speed (35,000 rpm). Probes were washed in 70% ethanol and rinsed in sterile PBS between samples. Once all lungs were homogenized, 20 μL was removed for CFU plating, described below, while the remainder was centrifuged at 16,000× *g* for 30 min at 4 °C then stored at −80 °C for subsequent cytokine and Western blot analysis.

### 2.7. Measurements of Bacterial Load in BALF and Lung Tissue

The obtained BALF and homogenized lung samples were aliquoted and serially diluted 1:10 in PBS. Lung homogenates were spot-plated on LB plates in 10 μL spots from 0 to 10^−3^ dilutions. Undiluted and 10^−1^ dilutions of BALF were spread-plated on LB plates. Afterward, plates were incubated upside-down at 37 °C overnight, and bacterial colonies were counted in the following morning. CFU was quantified using the following calculation: CFU/mL = (colonies × dilution factor)/volume plated (mL).

### 2.8. Cytokine Analysis in Lung Tissue, BALF and Serum

The levels of inflammatory cytokines, including interleukin-6 (IL-6), interleukin-1β (IL-1β), and tumor necrosis factor-α (TNF), were assessed by enzyme-linked immunosorbent assay (ELISA) according to the instructions provided by the manufacturer (R&D Systems, Minneapolis, MN, USA). Lung samples were diluted at 1:5 (IL-6, TNF) or 1:20 (IL-1β), and BALF and serum were diluted at 1:2.

### 2.9. Western Blotting

Western blotting analyses were performed as described previously [13]. Protein content was quantified via BCA assay (Pierce^TM^ BCA Protein Assay kit, Thermo Fisher Scientific, Waltham, MA, USA), and equal amounts of protein (40 μg/lane) for all samples were separated by 12% SDS-PAGE and transferred onto a polyvinylidene fluoride membrane (Millipore, Billerico, MA, USA). The primary antibody against NF-κBp65 (1:1000, Cell Signaling Technology, Danvers, MA, USA) was added and incubated overnight. Blots were then incubated with horse radish peroxidase-linked anti-rabbit immunoglobulin G (diluted 1:3000) for 2 h. With an enhanced chemiluminescence system (Chemidoc, Bio-Rad, Hercules, CA, USA), the protein of interest was detected, and the intensity of each band was analyzed using Image J V1.8.0 (NIH, Bethesda, MD, USA). GAPDH (1:2000, Cell Signaling Technology, Danvers, MA, USA) was defined as a loading control.

### 2.10. Histology

Mice were anesthetized by inhaled isoflurane 24 h post-infection as previously described and sacrificed by cervical dislocation. Lungs were collected and fixed in 10% neutral-buffered formalin (NBF) for 24 h and then washed and stored in 70% ethanol until processing. The fixed lung samples were sent to the Department of Pathology, IWK Health Centre, Halifax, NS, Canada, for further processing, including paraffin embedding, sectioning, and staining with hematoxylin and eosin. A blinded histological analysis was performed using an established lung injury score, including the presence of edema, hemorrhage, immune cell infiltration, cell wall thickening, and presence of vasculitis [15]. Representative histology images were taken using Optika Microscopes (Ponteranica BG, Italy).

### 2.11. Statistical Analysis

All data were analyzed using the software Prism 10 (GraphPad Software, La Jolla, CA, USA). To confirm the normal distribution of data, the Kolmogorov–Smirnov test was used. Pairwise comparisons were performed using Student’s *t*-test. One-way ANOVA or Kruskal–Wallis test was used to analyze multiple comparisons. Data were expressed as mean ± standard deviation (SD). Significance was assumed at *p* values less than 0.05 (*p* < 0.05).

## 3. Results

### 3.1. Effect of DIBI on Clinical Scores and Body Weight of Mice Infected by PA14

**Figure 1:**
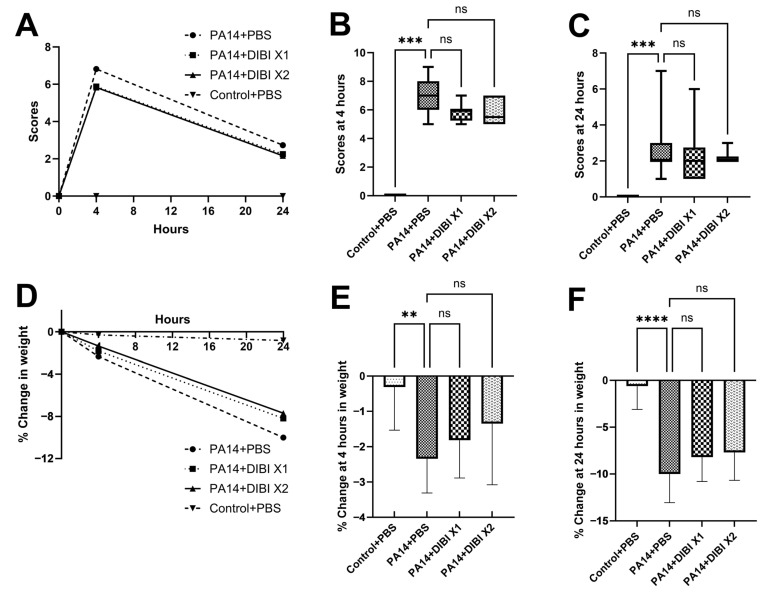
Changes in clinical scores and body weight of mice suffering from lung infection with *P. aeruginosa* PA14 over 24 h. (**A**–**C**) Representative of the change in clinical scores between groups at 4 and 24 h post-infection with *P. aeruginosa* strain PA14. (**D**–**F**) Representative of the change in body weight between groups at 4 and 24 h post-infection with *P. aeruginosa* strain PA14. Data were expressed as mean ± standard deviation and compared by Kruskal–Wallis test (**B**,**C**) or one-way ANOVA test (**E**,**F**). ** *p* < 0.01; *** *p* < 0.001; **** *p* < 0.0001. ns: no significant difference.

We assessed the clinical scores of mice 4 and 24 h after PA14 administration to observe the effects of DIBI treatment on the symptoms of infection at peak infection (4 h) and during the resolution phase (24 h). Lung infection with *P. aeruginosa* strain PA14 led to a significant increase in clinical score compared to the Control + PBS group. However, compared to the PA14 + PBS group, there was no difference in clinical scores with DIBI X1 or DIBI X2 treatment, indicating that DIBI treatment did not significantly affect clinical score (Figure 1B,C).

Body weight is considered an indicator of health, as mice experiencing symptoms of acute illness are unable to ambulate to reach food or water and thus lose body weight in a short period of time. Therefore, the impact of DIBI treatment on this read-out was recorded. Compared with the Control + PBS group, the body weight in the PA14 + PBS group decreased rapidly after infection. Although both groups of DIBI treatment mice lost less body weight at 24 h after PA14 infection, no significance was detected compared to the PA14 + PBS group (Figure 1E,F).

### 3.2. Two Dose of DIBI, but Not One Dose, Significantly Reduced Bacterial Growth in Mice Infected by PA14

**Figure 2:**
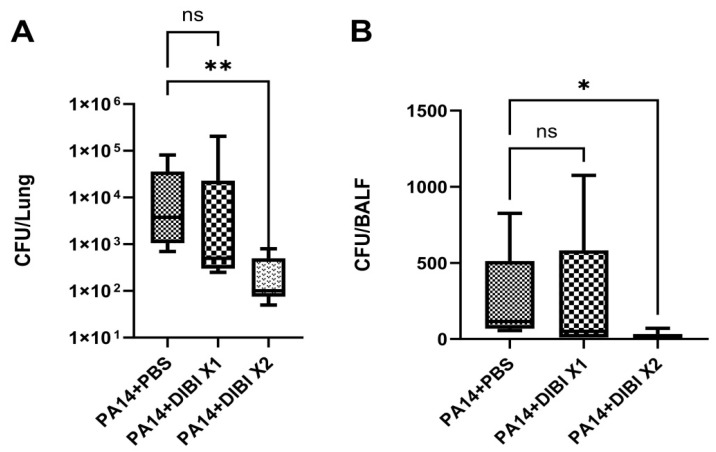
Effect of DIBI treatment on bacterial counts in lung homogenates (**A**) and bronchoalveolar lavage fluid (BALF, **B**) of mice suffering from *P. aeruginosa* PA14 infection. (**A**) Representative of the change in *P. aeruginosa* strain PA14 colony counts in lung homogenates. (**B**) Representative of the change in *P. aeruginosa* colony counts in BALF. Data were expressed as mean ± standard deviation and compared by Kruskal–Wallis test. * *p* < 0.05; ** *p* < 0.01; ns: no significant difference.

To evaluate the antibacterial effect of the iron chelator DIBI, *P. aeruginosa* colony counts from each group were performed on both lung homogenates and BALF (Figure 2). After a single dose of DIBI (PA14 + DIBI X1 group), no difference was observed compared to the PA14 + PBS group in both lung homogenates and BALF (Figure 2A,B). However, two doses of DIBI treatment (PA14 + DIBI X2 group) significantly reduced *P. aeruginosa* counts in both lung homogenates and BALF in comparison to the PA14 + PBS group (Figure 2A,B).

### 3.3. Two Dose of DIBI, but Not One Dose, Significantly Restricted Inflammatory Cytokine Production in the Lungs of Mice Infected by PA14

**Figure 3:**
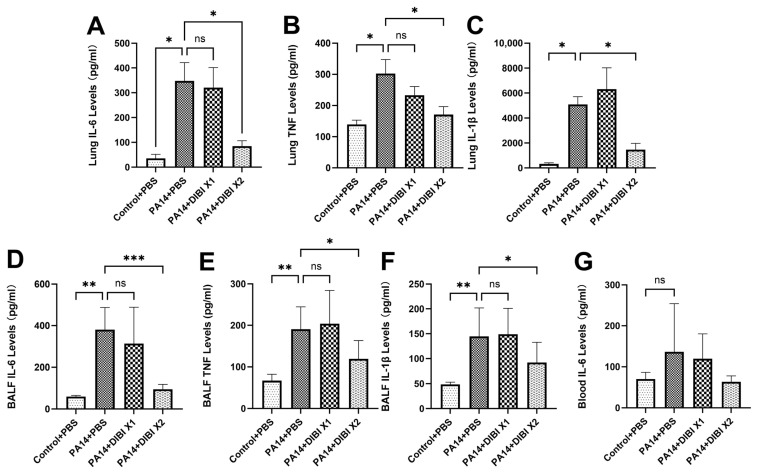
Effect of DIBI treatment on levels of inflammatory cytokines after *P. aeruginosa* PA14 infection. (**A**–**C**) Representative of changes in levels of inflammatory cytokines IL-6 (**A**), TNF (**B**), and IL-1β (**C**) in lung homogenates. (**D**,**F**) Representative of changes in inflammatory cytokines IL-6 (**D**), TNF (**E**) and IL-1β (**F**) in BALF. (**G**) Representative of changes in inflammatory cytokine IL-6 in serum. Data were expressed as mean ± standard deviation and compared by one-way ANOVA test. * *p* < 0.05; ** *p* < 0.01; *** *p* < 0.001; ns: no significant difference.

In order to evaluate the effect of DIBI on the inflammatory response after *P. aeruginosa* strain PA14 infection, we quantified the levels of inflammatory cytokine production in lung homogenates and BALF. As shown in Figure 3A–F, PA14 infection induced significant increases in IL-6, TNF and IL-1β in the PA14 + PBS group compared to the Control + PBS group in both lung and BALF. Single-dose DIBI treatment did not reduce the expression levels of IL-6, TNF and IL-1β in lung homogenates (Figure 3A–C) or in BALF (Figure 3D,E). However, two doses of DIBI treatment significantly decreased the levels of IL-6, TNF and IL-1β in lung homogenates compared with the PA14 + PBS group (Figure 3A–C). Similarly, IL-6 levels in BALF were significantly decreased in the PA14 + DIBI X2 group (Figure 3D). Only inflammatory cytokine, IL-6, was detected in blood, but no significant differences were observed (Figure 3G).

### 3.4. Effect of DIBI on Levels of NF-κBp65 in Mice Infected by PA14

**Figure 4:**
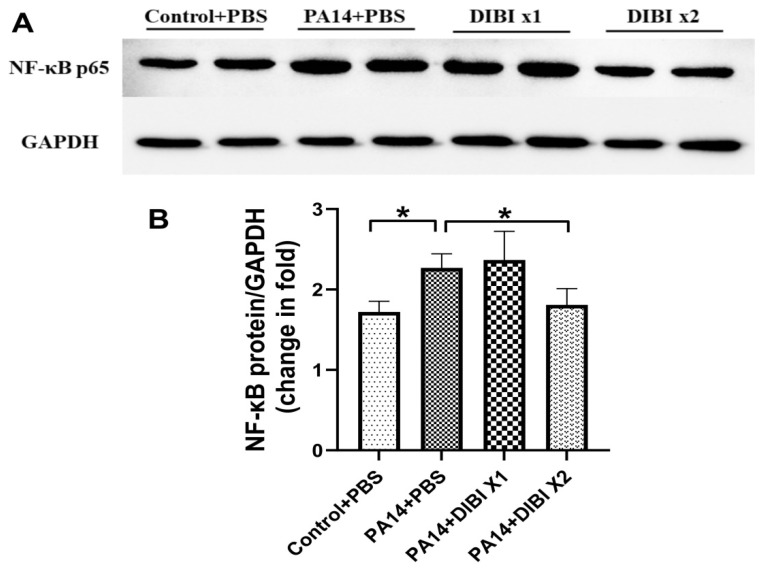
Effect of DIBI treatment on NF-κB levels in lung tissue after *P.* aeruginosa PA14 infection. (**A**,**B**) The protein levels of NF-κBp65 in lung tissue after *P.* aeruginosa PA14 infection. Data were expressed as mean ± standard deviation and compared by one-way ANOVA test. * *p* < 0.05.

To investigate the role of DIBI on levels of NF-κB after infection by *P*. *aeruginosa* PA14, the protein expression levels of NF-κBp65 in lung homogenates were assessed. As shown in Figure 4, compared with the Control + PBS group, PA14 infection induced increased NF-κBp65 expression, and two doses of DIBI treatment reduced the levels of NF-κBp65 in the PA14 + DIBI X2 group.

### 3.5. Effect of DIBI on Pulmonary Injury in Mice Infected by PA14

**Figure 5:**
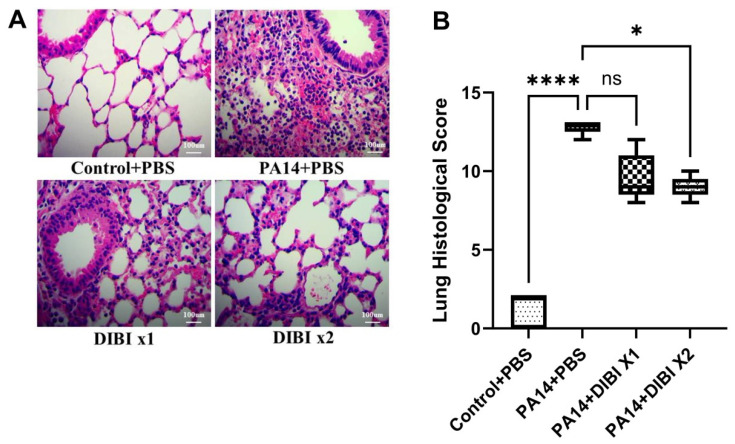
Effect of DIBI treatment on histological score of lung injury after *P. aeruginosa* PA14 infection. (**A**) Representative images of histopathologic changes in hematoxylin and eosin-stained lung sections at 400x magnification. (**B**) Histological injury was scored as described in the Materials and Methods section. Data were expressed as mean ± standard deviation and compared by Kruskal–Wallis test. * *p* < 0.05; **** *p* < 0.0001; ns: no significant difference.

Finally, we performed histological studies to determine the effects of DIBI on lung injury induced by *P. aeruginosa* PA14 infection. Compared to the Control + PBS group, PA14 infection led to severe histological lung injuries, including marked, usually diffuse and airway-centric, neutrophilic infiltration in the alveolar spaces and, to a lesser extent, in the alveolar walls, bronchiolar epithelium, and peribronchiolar soft tissue, along with moderate vasculitis in the associated vessels. In addition, we found some focal epithelial changes at the site of adjacent inflammatory cells, thickening of the peribronchial soft tissue, and reactive mesothelial cells in the PA14 + PBS group (Figure 5A,B). However, compared to the PA14 + PBS group, two doses of DIBI treatment resulted in a significant mitigation of the pulmonary histological changes following PA14 infection (Figure 5A,B). These findings suggest that in the model of acute lung infection induced by *P. aeruginosa*, two doses of DIBI administration can alleviate the severity of lung injury.

## 4. Discussion

With the present study, we revealed the role of iron chelator DIBI treatment on relieving some disease symptoms in an acute lung infection in the mouse model of *P. aeruginosa.* Specifically, the systemic administration of DIBI decreased the bacterial load in lung tissue and BALF, reduced pro-inflammatory cytokines production, and improved bacterial lung injury in histology.

Based on the fact that weight loss in *P. aeruginosa*-infected mice was associated with the inflammatory process, we also observed the effect of DIBI on weight change at 4 and 24 h after lung infection. Our results showed that the mice weight in the PA14 + PBS group reduced about 10% at the point of 24 h, which is consistent with other reports [16,17], thus suggesting that the mice suffered from an acute inflammatory response. In addition, although there was no statistically significant difference in weight loss between DIBI treatment and the PA14 + PBS group, our study found that DIBI-treated mice exhibited a tendency of less body weight decrease at both 4 and 24 h (Figure 1). After *P. aeruginosa* infection in mice, it was reported that weight loss was greatest at day 3 [17,18,19]. Rossi et al. reported in a model of *P. aeruginosa* lung infection that the body weight of the mice was monitored continuously for 6 days, but until the 4th day, β-sitosterol treatment exhibited significant faster body weight recovery [16]. Therefore, it would be of great interest to study the potential effect of DIBI on weight loss after *P. aeruginosa* lung infection at later time points.

In our experiment, we showed that after 24 h of lung infection induced by *P. aeruginosa*, double dose i.p. DIBI treatment was efficient in reducing bacterial load in both lung homogenate and BALF. In agreement with our findings on DIBI-restricted *P. aeruginosa* growth, it has previously been demonstrated that iron-withdrawal chelator DIBI inhibited the growth of *S. aureus* and alleviated the infection of mice caused by methicillin-resistant *S. aureus* (MRSA) [20]. In previous studies published by our group, we found that in sepsis induced by colon ascendens stent peritonitis (CASP), DIBI treatment significantly decreased the bacterial count in blood and peritoneal lavage fluid [21]. Consistently, it has been demonstrated that other iron chelators have the potential ability to combat *P. aeruginosa* biofilms. For example, Houshmandyar et al. found that deferiprone (DFP) can be inhibitory to the growth of *P. aeruginosa* with high concentration [22]. Similarly, gallium is also under consideration as an anti-pseudomonal agent, because it can inhibit *P. aeruginosa* growth and biofilm formation by disrupting bacterial iron homeostasis [23]. Taken together, our results indicate that with the anti-bacterial activity, DIBI could be a potential adjunct or alternative therapeutic approach for treating lung infections caused by *P. aeruginosa* by effectively limiting bacterial growth in vivo.

Because inflammation is a hallmark of lung infection, we also investigated the effect of DIBI administration on inflammatory mediators. The results demonstrated that treatment with two doses of DIBI significantly reduced cytokine levels including IL-6, TNF, and IL-1β in both lung homogenates and BALF. Similar to our results, other studies also showed that iron chelators inhibit the production of inflammatory cytokines. For example, Cheon et al. demonstrated that IL-6 and TNF production were completely decreased by i.p. deferoxamine (DFO) injection in rats [24]. In the model of lung ischemia reperfusion, Liu et al. showed that the levels of IL-6, TNF, and IL-1β were dramatically inhibited after DFO administration [25]. Interestingly, DFO has also been proven to alleviate viral replication and suppress consequent inflammatory cytokine storms, suggesting iron chelators can attenuate complications related to iron overload in diabetic patients with COVID-19 [26,27]. In association with the anti-inflammatory activity of DIBI, our study also showed that after treatment with two doses of DIBI, the protein abundance of NF-kB in lung tissues was reduced significantly. Consistent with our research, it was reported in other studies that LPS-induced NF-kB expression could also be inhibited by iron chelators, such as DFP and DFO [28,29]. In our previous experiments in vivo, it was confirmed that the levels of NF-κBp65 activation induced by LPS administration are reduced by DIBI treatment [13]. Therefore, as with DFO and other chelators, DIBI exhibited anti-inflammatory effects so as to decrease the acute lung injury induced by *P. aeruginosa.*

However, this study did not demonstrate a significant decrease in bacterial load and inflammatory mediator levels for treatment with one dose of DIBI. It is likely that our protocol utilized a low dose of bacterial inoculum (5 × 10^5^ CFU), as evidenced by our results showing that the highest clinical scores peaked at 7 (with 12 being the humane endpoint cutoff, for reference) at 4 h after *P. aeruginosa* administration. Therefore, the window for DIBI treatment is narrow to demonstrate a beneficial effect. Moreover, different from other reports [16,17,30], the *P. aeruginosa* inoculum was injected by way of the supraglottic region in our experiment, which would lead to more variability in the response to maximal inhalation of inoculum. Thirdly, the shorter half-life of DIBI has an effect on the duration of action of the drug, so without monitoring the blood concentration of DIBI, a single dose of DIBI may have limited efficacy.

For future studies, aerosolized or intravenous DIBI administration should be explored which could potentially be more effective in lung infection. In addition, as suggested by others [31,32], the effects of DIBI should be compared with standard antibiotics in lung infection induced by *P. aeruginosa*. An increasing body of evidence showed that DIBI as an adjunct to ciprofloxacin and other antibiotics could significantly improve antibiotic efficacy and reduce antibiotic resistance development [11,31,33]. Our current study demonstrated great potential for using DIBI to reduce bacterial growth and host inflammation, so it is of great interest to evaluate the benefits of combination therapy with other antibiotics in the model of lung infection induced by *P. aeruginosa*.

## 5. Conclusions

The present study observed anti-bacterial and anti-inflammatory effects of the iron chelator, DIBI, in an experimental model of lung infection induced by *P. aeruginosa* in mice. We found that two doses of DIBI administration reduced bacterial load and decreased NF-κB activation and inflammatory mediator release, so as to attenuate lung histological injury. These results suggest that DIBI acts as a potent multi-targeting agent able to break the vicious cycle of inflammation induced by pathogens and improve outcomes in *P. aeruginosa*-related lung pathology and sequelae.

## Data Availability

The raw data supporting the conclusions of this article will be made available by the authors on request.
